# Nursing students' perceptions of an anti-stigma intervention for schizophrenia: a qualitative focus group study

**DOI:** 10.3389/fpubh.2026.1870693

**Published:** 2026-06-25

**Authors:** Jing Wang, Hong Wang, Xi Chen

**Affiliations:** 1 Department of Obstetrics & Gynecology, The First People's Hospital of Foshan (Foshan Hospital Affiliated to Southern University of Science and Technology), School of Medicine, Southern University of Science and Technology, Shenzhen, Guangdong, China; 2 Department of Anesthesiology, The First People's Hospital of Foshan (Foshan Hospital Affiliated to Southern University of Science and Technology), School of Medicine, Southern University of Science and Technology, Shenzhen, Guangdong, China; 3 School of Nursing, The Hong Kong Polytechnic University, Kowloon, Hong Kong, SAR China

**Keywords:** anti-stigma intervention, focus group interviews, nursing students, qualitative research, schizophrenia, social stigma

## Abstract

**Aim:**

To explore nursing students' experiences of participating in a schizophrenia stigma-reduction intervention and identify implications for intervention refinement and implementation.

**Background:**

Nursing students play a critical role in the care and rehabilitation of individuals with schizophrenia. However, their experiences with stigma-reduction interventions remain underexplored, limiting evidence to optimize intervention design and delivery.

**Design:**

A qualitative descriptive study.

**Methods:**

Purposive sampling was used to recruit nursing students who had participated in a pilot randomized controlled trial (RCT) conducted in Changsha, Hunan Province, China (22–27 March 2023). Data were collected through online focus group interviews via the Tencent Video Conference platform. Data collection and analysis were conducted concurrently, using expanded field notes, coding tables, and iterative categorization to identify themes.

**Results:**

Thirty nursing students participated in five focus group interviews (4–8 participants per group; 46–113 min; mean ≈ 68 min). Participants were aged 20–23 years; 77% were female. Four themes emerged: feasibility of the intervention, accessibility of the intervention, perceived efficacy of the intervention, and suggestions for future implementation.

**Conclusions:**

The intervention was perceived as feasible, accessible, and effective by nursing students. Participants also provided practical recommendations to improve intervention design and delivery. These findings offer valuable insights to inform the optimisation and future implementation of schizophrenia stigma-reduction interventions among health professional students.

## Introduction

Schizophrenia affects approximately 24 million individuals globally, corresponding to 0.32% of the population, or roughly 1 in 300 individuals. Among adults, the estimated prevalence rate is about 0.45% of the population, equivalent to around 1 in 222 individuals (1). In China, the lifetime prevalence of schizophrenia is estimated at 0.6%, affecting approximately 7 million individuals, representing a substantial public health burden (2).

Stigma is a complex social process involving the labeling, stereotyping, separation, status loss, and discrimination of individuals possessing a socially devalued attribute within a context of unequal power relationships (3). Individuals with schizophrenia commonly encounter stigma across multiple domains of life, including employment (4), interpersonal and romantic relationships (4, 5), rehabilitation services (6), community participation (6), and healthcare settings (7). These stigmatizing experiences can have far-reaching consequences, including reduced social and occupational opportunities, difficulties establishing and maintaining meaningful relationships, social exclusion, delayed help-seeking and treatment utilization, reluctance to disclose mental health concerns, symptom neglect, loss of self-esteem, poorer quality of life, and ultimately poorer recovery outcomes (8, 9).

The effects of schizophrenia-related stigma extend beyond individuals with the condition to those who are closely associated with them. Family caregivers often experience courtesy stigma (also known as associative stigma), whereby they are negatively judged, blamed, or socially excluded because of their relationship with a person with schizophrenia (10). Such stigma may manifest through social distancing, criticism of caregiving practices, or assumptions that family members are responsible for the illness, contributing to emotional distress, caregiver burden, and reduced social support (11). Similarly, mental health professionals working in psychiatric settings may encounter stigma by association, as their close involvement with individuals with mental illness can lead to negative perceptions from colleagues, patients, and the wider public (12). These stigmatizing attitudes may reduce professional prestige and job satisfaction, potentially contributing to workforce shortages in mental health services (13).

Stigmatization arises from factors such as inadequate knowledge, prejudicial attitudes, and discriminatory behavior (14). Nurses are the largest group of healthcare workers globally and are responsible for the care and recovery of individuals with schizophrenia (15, 16). Attitudes developed during nursing education may persist throughout professional careers and directly influence future patient care. Within mainland China, nursing students exhibit a passive attitude toward individuals with mental disorders (17, 18) and possess lower levels of knowledge regarding schizophrenia compared to their counterparts in the USA (19). Understanding nursing students' attitudes toward schizophrenia is crucial because these attitudes may directly influence the quality of future mental health care delivery. Previous research suggests that educational, contact-based, and simulation interventions can improve nursing students' knowledge, attitudes, and empathy toward people with mental illness, which may contribute to more compassionate and person-centered care in future clinical practice (20–22). Altering nursing students' negative attitudes and improving their knowledge of schizophrenia may reduce stigmatization of individuals with this condition. These improved attitudes may persist throughout their nursing careers. Stigma within healthcare settings is particularly concerning because it directly influences the quality of care received by individuals with schizophrenia.

Stigmatization of mental illness is influenced by various factors, with cultural factors playing a significant role (23). Across different cultures, there exist varying interpretations of stigma, distinct approaches to its management, and diverse outcomes (24). Chinese culture, deeply rooted in Confucianism, Taoism, and Buddhism, can lead individuals to respond uniquely when confronted with schizophrenia (25, 26). In Chinese culture, mental illness may be associated with family shame, moral judgment, or beliefs related to karma, which can shape both public attitudes and healthcare professionals' responses toward individuals with schizophrenia (27).

Previous anti-stigma interventions for nursing students have primarily focused on improving knowledge and attitudes through education, contact-based learning, and simulation (21, 22, 28). However, most interventions were developed in Western contexts and may not adequately address the cultural beliefs influencing stigma in Chinese settings. Although a pilot randomized controlled trial suggested that the Chinese-culture-specific intervention may be beneficial, little is known about participants' experiences, perceived acceptability, feasibility, and recommendations for improvement (27). Understanding these experiences is essential for refining future interventions.

A qualitative descriptive approach was adopted to explore nursing students' experiences of participating in the intervention. This approach is appropriate for obtaining rich descriptions of participants' perceptions, experiences, and suggestions within a real-world context and is well suited for understanding participants' subjective experiences (29, 30). Findings from this study will inform the refinement and future implementation of culturally tailored anti-stigma interventions for nursing students and other healthcare professional students. Therefore, this study aimed to explore nursing students' experiences of participating in a Chinese-culture-specific intervention designed to reduce schizophrenia stigma and to identify suggestions for improving future intervention development and implementation.

### Ethical issues and data safety

This study was approved by the Research Ethics Committee of The Hong Kong Polytechnic University (HSEARS20220127002, approved on 22 February 2022) and the Research Ethics Committee of Xiangya Hospital (KE202203129, approved on 18 March 2022). This study was not formally preregistered. However, it was conducted as part of a PhD research project, and the study design, data collection procedures, and analytical approach were specified in the approved doctoral research protocol prior to data collection. Potential participants who had completed the intervention training were invited to provide online informed consent before participating in this study. They were informed that participation was entirely voluntary and that they could withdraw from the study at any time without any adverse consequences. Participants were approached by the researcher only after they had read the online informed consent form and indicated their willingness to participate. Silence or non-response from potential participants was not regarded as consent to participate. Before each focus group interview, the principal investigator explained the purpose and significance of the study. All study data were kept confidential, and access was restricted to authorized members of the research team only. The data will be securely stored and deleted 3 years after the completion of the study. Each participant received 50 Chinese yuan as a token of appreciation for their time and participation in the focus group interview.

### Theoretical framework guiding this study

In this study, the Socio-Ecological Model was used to guide the interpretation of the findings (31). This model emphasizes that nursing students' perceptions of the anti-stigma intervention for schizophrenia are shaped by multiple factors, including their personal beliefs, educational experiences, cultural values, broader social attitudes toward mental illness, and the stigma-reduction intervention they received.

Consistent with the interpretivist approach, this study recognizes that nursing students' perceptions of schizophrenia and the anti-stigma intervention are socially and culturally shaped. Values rooted in Chinese culture, such as family responsibility, moral judgment, and beliefs related to karma, may influence their attitudes toward individuals with schizophrenia. Therefore, the Socio-Ecological Model provides an appropriate framework for understanding these influences and the perceived effects of the intervention.

### Ontological, epistemological, axiological, and methodological foundations of the study

**Ontology:** This study adopts a relativist ontology, recognizing that nursing students' perceptions of schizophrenia and the anti-stigma intervention are shaped by their individual experiences and sociocultural contexts, and that multiple realities may exist.

**Epistemology:** An interpretivist epistemology is employed, whereby knowledge is understood as being co-constructed through interactions between researchers and participants during the focus group discussions.

**Axiology:** This study acknowledges that participants' attitudes toward schizophrenia may be influenced by personal values and Chinese cultural beliefs, while researchers' perspectives may also affect data interpretation. Therefore, reflexivity was maintained throughout the research process.

**Methodology:** Grounded in an interpretivist paradigm, this study adopts a qualitative descriptive design to explore nursing students' perceptions and experiences of a Chinese-culture-specific anti-stigma intervention for schizophrenia.

## Methods

This study report follows the reporting guidelines of the consolidated criteria for reporting qualitative studies (COREQ) 32-item checklist (32).

### Study design and setting

This study employed a qualitative descriptive approach to explore the experiences of nursing students who participated in an intervention aimed at reducing schizophrenia stigma. The study was conducted at a leading tertiary hospital in mainland China. Data were collected through focus group interviews conducted on Tencent Meeting, an online interview platform widely used in China. Focus group interviews were selected because they facilitate group interaction and generate richer, more comprehensive data through shared discussion and reflection (33). These interactions helped participants express their experiences of the intervention and provide suggestions for future intervention development. Through focus group discussions, the research team was able to collect individual perspectives while also encouraging interactive verbal exchanges among participants, which often led to new insights and deeper understanding. This approach also enabled the researchers to better understand participants' needs, emotions, and the influence of cultural values and beliefs on their perceptions (34). As focus groups are particularly suitable for exploring in-depth experiences within a specific population and for informing intervention refinement (33), focus group interviews were conducted between 22 March 2023 and 27 March 2023.

### Participants and recruitment

All participants in this study were fourth-year nursing students who had previously participated in the experimental group of the pilot randomized controlled trial (RCT). Recruitment was conducted through WeChat, with the principal investigator responsible for identifying eligible participants and obtaining informed consent. As all participants were recruited from the previous pilot RCT, they had already met the original eligibility criteria, including being aged 18 years or older and being able to communicate proficiently in Mandarin. Therefore, the inclusion and exclusion criteria for this qualitative study were defined as follows:

#### Inclusion criteria

(1) Nursing students who had participated in the experimental group of the previous pilot RCT. (However, the authors neither implemented the intervention nor established a relationship with participants.)

#### Exclusion Criteria

(1) Nursing students who were unwilling to participate in the study.

### Sampling

Purposive sampling was used to recruit participants with direct experience of the intervention.

### Sample Size

A recommended range of 4–12 participants is generally suggested for focus group interviews. However, managing more than 12 participants in an online focus group setting can be challenging. Therefore, participants were divided into two or three separate focus groups to improve manageability and facilitate effective discussion (35), with each group consisting of approximately six participants, as recommended in previous studies (34). To avoid inefficient use of time and resources, data collection was guided by the principle of data saturation, defined as the point at which no new codes or themes emerged from the interviews (36). As the final sample size depended on the achievement of data saturation, it could not be determined in advance (37). However, since the experimental group of the pilot randomized controlled trial (RCT) included 30 participants, the maximum sample size for this study did not exceed 30.

### Data collection

A literature review and research group discussions informed the development of the interview guide. Before the formal focus group interviews, two fourth-year nursing students who had not participated in the previous pilot RCT were invited to review and discuss the interview guide to improve its clarity and relevance. In addition, one assistant professor specializing in psychiatric nursing and one senior nurse working in the psychiatric department were invited to provide expert comments on the guide. Based on their feedback, the principal researcher revised the interview guide, and the research team conducted further discussions to finalize it. Details of the interview guide are provided in Appendix 1.

Tencent Meeting was used to visually conduct and record the online focus group interviews. A sociodemographic questionnaire was used to collect participants' demographic information, and the results are presented in Table 1. The lead researcher, a male third-year PhD candidate with postgraduate research training, conducted all focus group interviews. Meanwhile, a research assistant recorded participants' non-verbal cues and emotional responses through field notes.

**Table 1:** Detailed information on the sample characteristics.

Demographic characteristics	Number (%)
Gender	
Female	23 (76.67)
Male	7 (23.33)
Age (Year)	
20-21	15(50.00)
22-23	15 (50.00)
Ethnicity	
Han	27 (90.00)
Others	3 (10.00)
Marital state	
Married	0
Unmarried	30 (100.00)
Religion Belief	
Yes	2(6.67)
No	28(93.33)

Given the online format of the interviews, participants were allowed to choose a comfortable and private setting for participation. Introductory questions were used at the beginning of each session to establish rapport and encourage open discussion. All interviews were audio-visually recorded and fully transcribed. To ensure transcript accuracy, the research assistant and the lead researcher independently cross-checked the transcripts against the recorded videos and compared the two versions to identify discrepancies. Any differences were resolved through joint review of the recordings to produce the final transcript. The finalized transcripts were then returned to participants for member checking, allowing them to clarify inconsistencies and improve data accuracy. Non-verbal responses were also documented in the transcripts in accordance with the guidance of Creswell (38).

### Data analysis

Data analysis began immediately after the interview transcripts were completed, and coding was initiated promptly. The lead researcher read the transcripts several times to become familiar with the data and facilitate subsequent analysis. Demographic characteristics of the participants were summarized using frequencies and percentages.

Interview transcripts were analyzed using inductive qualitative content analysis, examining both manifest and latent content (39–41). Coding was conducted inductively from the interview data, while the Socio-Ecological Model informed the interpretation of the findings rather than the coding process.

Recognizing that concurrent data collection and analysis can improve the depth and quality of qualitative research (42). Interviews continued until no new codes, categories, or themes emerged.

The analysis followed five stages: data preparation and organization, repeated reading and memoing, coding, category generation, and theme development (38). Two researchers independently analyzed the transcripts and compared their coding results. Any discrepancies were resolved through discussion until consensus was reached, and the final themes and subthemes were agreed upon accordingly. In addition, an experienced qualitative researcher reviewed the analysis process and assessed the consistency between the original data and the identified themes, further enhancing the rigor of the study.

Data analysis was conducted manually using extended tables, large sheets of paper, and colored markers. First, two copies of each transcript were prepared and organized systematically. The transcripts were then read and reread to gain a comprehensive understanding of the data. Large sheets of paper were prepared for each interview question or potential theme, and different colored markers were used to highlight quotations related to possible subthemes. The highlighted quotations were then cut out and placed onto the corresponding sheets. Finally, the quotations were compared, summarized, and grouped into relevant subthemes and themes to identify their underlying meanings.

To improve credibility, the preliminary findings were returned to participants for feedback and clarification. All analyses were first conducted in Chinese. After the findings were finalized, they were translated into English. The translation was reviewed by an English language teacher to minimize any loss of meaning and ensure consistency with the original Chinese data.

### Issues of data trustworthiness

To ensure the trustworthiness of this qualitative study, four criteria were considered: credibility, dependability, transferability, and confirmability (43). Credibility was enhanced through peer debriefing, member checking, and the transcription of all interview data into Chinese by an independent research assistant. After data analysis, categories and subcategories were developed, and the findings were translated into English to ensure consistency and minimize loss of meaning during translation (44).

Dependability was supported by maintaining a comprehensive audit trail and analytical memos to document each stage of the research process. Transferability was strengthened by providing detailed descriptions of participants' demographic characteristics and the study context (45).

To ensure confirmability, the researcher engaged in reflexivity to identify any unintentional influence on participants' responses. A research assistant also reviewed the video recordings to identify potential interviewer bias. In addition, peer debriefing was conducted with two experienced research assistants who reviewed the analytical process and interpretation of findings, further supporting the confirmability of the study (46).

## Results

### Demographic and characteristics of participants

Between March 22 and March 27, 2023, five focus group interviews were conducted with 30 fourth-year nursing students following completion of the fourth week of intervention training, with no participant dropout. Data saturation was achieved by the fifth focus group interview, as no new themes or subthemes emerged during the final interview. No participants expressed disagreement with the interpretation of the results. Detailed demographic characteristics of the participants are presented in Table 1.

All interviews were conducted online using the Tencent Meeting platform. Each session was audio-recorded in full and transcribed verbatim to ensure data accuracy and completeness. No repeat interviews were required, as each focus group generated sufficient depth and richness of information. The five focus groups consisted of 7, 8, 6, 5, and 4 participants, respectively. Relatively small group sizes were intentionally maintained to facilitate deeper discussion and encourage participants to share detailed reflections, particularly given that they had completed 4 weeks of intervention training and were able to provide meaningful insights based on their experiences.

The duration of the focus group interviews ranged from 46 to 113 min, with an average duration of approximately 68 min.

Four major themes were identified from the thematic analysis: (1) feasibility of the intervention, (2) accessibility of the intervention, (3) perceived efficacy of the intervention, and (4) suggestions for future intervention implementation. Overall, participants reported positive experiences regarding the intervention's structure, delivery mode, and perceived effectiveness in reducing schizophrenia stigma, while also providing practical recommendations for improving future implementation. The coding tree and thematic structure are presented in Table 2.

**Table 2:** Coding tree of the focus group interview findings.

Theme	Sub-theme	Code units
Feasibility of the intervention	The duration and frequency of the intervention were appropriate	Frequency and time costing acceptable
	The time taken for the intervention reports was appropriate	Reporting time was suitable
	The online intervention delivery was appropriate	Online intervention was good
		Online intervention was convenient
		Like online intervention
Acceptability of the intervention	Intervention was well to accept	Like the intervention content
		Feel the novelty of the intervention
		The intervention makes sense
		The content of the intervention was well accepted
		Feeling like the intervention paid off
		Intervention was easy to complete
	Measuring tools were appropriate	Moderate number of scales
		Scales were suitable for measuring the intervention effects
		understand the content of the scale
The effect of the intervention	Knowledge increase	Acquire correct knowledge
		Recognize that stigma exists
	Negative attitude decrease	Change in attitude
		Change in stereotype
	Positive coping behavior	Treat well
		Know how to cope
		Correct wrong thinking of others
		Non-discrimination
		Won't make fun of
		Willing to work in psychiatry department
	Empathy improvement	Increased empathy
		From the patient's point of view
		Understand people with schizophrenia
Recommendations for future interventions	Sections of intervention which can be improved	Teaching students to draw concept maps
		Limit reporting time and reduce duplication
		Reduce workload
		Extended preparation time
		Increase participant communication
		Intervention changed to face to face
		Election of the team leader, a person responsible for
		Everyone reports their own content separately
		Prepare well to intervene
		Adjust the sequence of interventions
		Unpack content that is too interfering
		Increase the number of contacts in the intervention
	Intervention measurement tools selection	Increase scales number
		Choose a scale that can be measured in more depth

To facilitate understanding of the findings of this qualitative study, a brief description of the intervention is provided below.

### Description of the intervention

Participants had previously completed a 4-week Chinese culture-specific schizophrenia stigma-reduction intervention as part of a pilot randomized controlled trial. The intervention was developed to improve nursing students' knowledge of schizophrenia, correct common misconceptions, enhance empathy, and reduce stigmatizing attitudes and behaviors. It incorporated educational, contact-based, reflective, and collaborative learning strategies delivered through four sequential weekly modules.

During Week 1, students learned about the etiology, clinical manifestations, treatment, prognosis, and legal issues related to schizophrenia through investigative, interactive, and higher-order learning activities. Week 2 focused on identifying and critically evaluating inaccurate portrayals of schizophrenia in social media, literature, films, and television. In Week 3, students engaged in direct contact with individuals recovering from schizophrenia and psychiatric nurses, enabling discussion of lived experiences and challenging common stereotypes. Week 4 emphasized self-reflection on stigma through discussions of traditional Chinese cultural and religious beliefs, reflective writing exercises, and collaborative concept-mapping activities. The intervention was delivered in small groups and combined investigative learning, interactive discussion, collaborative learning, and critical reflection. Additional details of the intervention are provided in Appendix 2.

### Cultural adaptation of the intervention

The intervention was culturally tailored to the Chinese context in several ways. First, educational content addressed traditional Chinese cultural and religious beliefs that may influence perceptions of schizophrenia, including perspectives derived from Confucianism, Taoism, and Buddhism. Second, participants critically examined stigmatizing portrayals of schizophrenia in Chinese social media, literature, films, and television programmes. Third, reflective activities encouraged students to consider how Chinese cultural values, religious beliefs, and societal attitudes may contribute to stigma toward individuals with schizophrenia. Finally, contact-based sessions with people recovering from schizophrenia and psychiatric nurses provided opportunities to challenge culturally influenced misconceptions through direct interaction and discussion.

### Theme 1: Feasibility of the intervention

This theme reflected participants' perceptions of the practicality and manageability of the intervention and consisted of three sub-themes: (a) appropriate duration and frequency of the intervention, (b) appropriate time required for intervention reporting, and (c) suitability of online intervention delivery.

#### Sub-theme 1: Appropriate duration and frequency of the intervention

Participants generally considered the intervention frequency and duration to be appropriate and manageable within their academic and clinical schedules. Weekly intervention sessions were viewed as optimal, as they maintained continuity without creating excessive burden. Participants reported that longer intervals between sessions could reduce retention and engagement. As one participant stated:

*I don't think there is any problem with the frequency of the intervention. If the intervention is just once a week, it's actually fine. Once a week is equivalent to having a small task per week; it is not difficult*. (Participant 3)

Another participant added:

*Once a week is okay, but once every two weeks is too long, and it's easy to forget*. (Participant 16)

These responses suggest that a moderate and regular intervention schedule supported participant adherence and learning.

#### Sub-theme 2: Appropriate time required for intervention reporting

Participants also reported that the time allocated for reporting and reflection during the intervention was appropriate. Most participants considered approximately 2 h per session acceptable and sufficient for completing the required tasks without excessive fatigue.

One participant explained:

*The time for each report is about 2 hours, and I feel that the duration is good*. (Participant 26)

Another participant noted:

*Too much content at once may not be good. I feel that 2 hours for the report was fine*. (Participant 27)

These findings indicate that maintaining a manageable reporting workload contributed positively to the intervention's feasibility.

#### Sub-theme 3: Suitability of online intervention delivery

Participants considered the online delivery format highly convenient and practical, particularly during clinical placement periods. Online sessions reduced travel time and allowed flexible participation, which improved accessibility and reduced disruption to daily routines.

One participant commented:

*Online intervention was very good. I can listen to lectures and eat at the same time. I find it very convenient and much better than face-to-face*. (Participant 11)

Another participant stated:

*I think online intervention is very good. The benefits far outweigh the disadvantages*. (Participant 3)

These findings indicate that online intervention delivery contributed positively to the feasibility of the intervention.

### Theme 2: Accessibility of the intervention

This theme described participants' acceptance of the intervention and the suitability of the measurement tools and included two sub-themes: (a) high acceptability of the intervention and (b) appropriateness of the measurement tools.

#### Sub-theme 1: High acceptability of the intervention

Participants reported that the intervention was easy to accept and that their acceptance increased as they became more familiar with the process. Direct communication with psychiatry nurses and people recovering from schizophrenia was perceived as particularly meaningful and authentic.

One participant shared:

*I liked to communicate directly with the psychiatry nurses and people recovering from schizophrenia. It felt real to me*. (Participant 1)

Another participant noted:

*I believe it's beneficial for research acceptance because it felt more intuitive and meaningful*. (Participant 7)

These findings suggest that real-life interaction and experiential learning improved intervention acceptability.

#### Sub-theme 2: Appropriateness of the measurement tools

Participants considered the assessment scales appropriate, understandable, and easy to complete. They reported that the questionnaire length was acceptable and that the content was clear and relevant to the intervention outcomes.

One participant stated:

*The scales were very suitable; the content was not too much, just enough to fill them out carefully*. (Participant 26)

Another participant added:

*There is basically no problem with understanding these scales*. (Participant 6)

This suggests that the selected measurement tools were acceptable for evaluating intervention effectiveness.

### Theme 3: Perceived efficacy of the intervention

This theme reflected participants' perceived changes following the intervention and consisted of four sub-themes: (a) increased knowledge, (b) reduced negative attitudes, (c) improved behavioral intentions, and (d) enhanced empathy.

#### Sub-theme 1: Increased knowledge

Participants reported a better understanding of schizophrenia and mental illness after completing the intervention. Increased knowledge helped reduce uncertainty and improved confidence when interacting with people experiencing schizophrenia.

One participant stated:

*The biggest gain was a better understanding of people with schizophrenia*. (Participant 20)

Another participant explained:

*I feel that I have a more comprehensive understanding of schizophrenia*. (Participant 23)

#### Sub-theme 2: Reduced negative attitudes

Participants described a clear reduction in fear, resistance, and stereotypes toward people with schizophrenia. Many participants reported that their previous perceptions had been strongly influenced by media stereotypes.

One participant reflected:

*I used to think mental illness was associated with violence, but now I feel mental illness is not so scary*. (Participant 22)

Another participant stated:

*I am not as resistant and afraid to contact people with mental illness now*. (Participant 5)

#### Sub-theme 3: Improved behavioral intentions

Participants reported more positive intentions toward interacting with and supporting people with schizophrenia. They also described greater willingness to challenge stigmatizing language and discriminatory behaviors in daily life.

One participant stated:

*I will be more careful with my words and avoid making jokes about mental illness*. (Participant 11)

Another participant shared:

*If I were assigned to work in the psychiatric department, I wouldn't be so afraid. In fact, I still yearn to work there*. (Participant 15)

#### Sub-theme 4: Enhanced empathy

Participants described stronger emotional understanding and compassion toward people with schizophrenia. Listening to recovery stories and interacting with recovered patients helped them develop empathy and a greater sense of social responsibility.

One participant explained:

*I feel that I have more empathy towards people with schizophrenia now*. (Participant 7)

Another participant stated:

*I developed stronger empathy when I interacted with individuals who had recovered*. (Participant 10)

These findings suggest that empathy-building was an important mechanism underlying stigma reduction.

### Theme 4: Suggestions for future intervention implementation

This theme captured participants' recommendations for improving the intervention and included two sub-themes: (a) recommendations for optimizing intervention delivery and (b) recommendations for improving measurement tools.

#### Sub-theme 1: Recommendations for optimizing intervention delivery

Participants suggested that the first week contained excessive content and required substantial preparation time. Some recommended simplifying concept maps, reducing repetition, and improving peer interaction during group activities.

One participant stated:

*The content of the first week is a bit too much, and some topics are repeated*. (Participant 12)

Another participant suggested:

*If the concept map could be changed into a short video, students may find it easier to understand*. (Participant 3)

These suggestions indicate that improving instructional design may further enhance intervention engagement.

#### Sub-theme 2: Recommendations for improving measurement tools

Some participants suggested that the assessment scales could be more in-depth and better capture nuanced changes in schizophrenia-related knowledge and attitudes.

One participant stated:

*The questions in the scales can be a little more in-depth. I feel they are somewhat superficial*. (Participant 8)

Another participant noted:

*The answers related to schizophrenia are rather vague and could be more comprehensive*. (Participant 12)

These findings suggest that future studies may benefit from refining measurement tools to improve sensitivity and depth of outcome assessment.

## Discussions

This qualitative study explored the experiences of fourth-year nursing students who participated in a culturally specific Chinese intervention designed to reduce schizophrenia stigma. The findings demonstrated that the pilot randomized controlled trial (RCT) showed good feasibility, accessibility, and perceived effectiveness, while also providing important suggestions for improving future intervention implementation. These findings offer valuable evidence for optimizing stigma-reduction interventions among nursing students and support the design of future full-scale RCTs.

Theme 1 showed that participants considered the intervention highly feasible, particularly regarding its frequency, duration, and online delivery format. Appropriate intervention intensity is essential for maintaining participant engagement and maximizing intervention outcomes. Previous studies have shown that excessively frequent sessions or prolonged intervention periods may reduce adherence and increase participant burden, whereas overly brief or infrequent interventions may fail to produce meaningful behavioral change (47, 48). In this study, participants reported that the intervention schedule was manageable and compatible with their demanding clinical placement responsibilities, suggesting that the balance between intervention intensity and participant burden was appropriate. This finding is particularly important for nursing students, whose irregular clinical schedules often limit participation in traditional face-to-face programs.

The online delivery mode further enhanced feasibility by providing flexibility, convenience, and cost-effectiveness. Participants could access the sessions through smartphones at their preferred time and location, which reduced logistical barriers and facilitated sustained participation. This is consistent with previous studies showing that digital delivery improves accessibility and scalability, particularly during public health emergencies such as the COVID-19 pandemic and among populations with limited access to in-person education. From an implementation science perspective, online interventions may improve reach and sustainability while minimizing institutional resource demands. However, participants also reported that online delivery allowed multitasking and reduced real-time engagement, which may weaken intervention fidelity. This suggests that future studies should consider hybrid models that combine the flexibility of online learning with the interaction and accountability of face-to-face communication.

Theme 2 highlighted the high accessibility and acceptability of the intervention. Acceptability is a key determinant of intervention success and reflects the extent to which participants perceive an intervention as appropriate, engaging, and relevant to their needs (49, 50). In this study, participants reported that both the intervention content and the outcome measurement tools were understandable, relevant, and easy to accept. This may be partly explained by the cultural adaptation of the intervention, which improved contextual relevance and reduced resistance to discussing mental illness stigma. In Chinese cultural contexts, stigma related to schizophrenia is often strongly influenced by stereotypes, family shame, and social distancing. Therefore, culturally sensitive intervention content may be particularly important for promoting openness and reducing defensive attitudes.

High acceptability is closely associated with better adherence and stronger intervention effects (49, 51). Conversely, interventions with poor acceptability are less likely to be implemented as intended and may compromise overall effectiveness (52). The findings suggest that the pilot intervention successfully aligned with participants' educational expectations and social context, providing strong support for its use in future larger-scale trials. This also indicates that stigma-reduction interventions should move beyond knowledge transmission alone and incorporate culturally meaningful content that resonates with participants' lived experiences and professional identity development.

Theme 3 demonstrated that participants perceived the intervention as effective in improving knowledge, attitudes, intentional behaviors, and empathy toward people with schizophrenia. These findings are particularly important because stigma is a multidimensional construct involving cognitive, affective, and behavioral components. Improvements across these domains suggest that the intervention may have addressed stigma more comprehensively than traditional knowledge-based education alone. Previous studies have shown that educational interventions combined with role-play and direct or indirect social contact can significantly reduce mental illness stigma among nursing students (53). Consistent with contact theory, meaningful exposure to the lived experiences of individuals with schizophrenia can reduce fear, challenge stereotypes, and promote empathy.

Unlike previous interventions that mainly focused on general mental illness stigma, this study specifically incorporated cultural understanding and empathy-building strategies to address schizophrenia-related stigma. Participants reported greater understanding of schizophrenia, reduced fear when interacting with affected individuals, and changes in stigmatizing behaviors, such as avoiding discriminatory jokes and showing greater willingness to work in psychiatric settings. These findings suggest that empathy may serve as an important mechanism linking knowledge acquisition to behavioral change. This is consistent with attribution theory, which proposes that reducing blame and increasing understanding of illness causes can reduce prejudice and discriminatory responses. Therefore, future stigma-reduction interventions should strengthen empathy-based learning components rather than relying solely on didactic education.

Theme 4 revealed that participants provided valuable suggestions for improving intervention delivery and outcome assessment. Some participants preferred face-to-face sessions because they facilitated interaction, emotional engagement, and peer discussion, which are particularly important when addressing sensitive topics such as stigma and prejudice. Others suggested improving the evaluation tools to better capture subtle attitudinal and behavioral changes. These recommendations highlight the importance of participant-centered intervention refinement. There is no universally optimal intervention model for stigma reduction, and intervention effectiveness may depend heavily on contextual adaptation and participant preferences.

Qualitative feedback provided insights that could not be fully captured through quantitative outcome measures alone. While the pilot RCT demonstrated preliminary effectiveness, the focus group interviews helped identify practical barriers such as preparation burden, preferred delivery methods, and measurement limitations. These findings strengthen the overall rigor of the pilot study by explaining not only whether the intervention worked, but also how and why it worked. This integration of qualitative and quantitative evidence is particularly valuable for refining complex behavioral interventions before conducting definitive full-scale RCTs.

## Limitations

Several limitations should be considered when interpreting the findings. First, focus group interviews were used for qualitative data collection, which may have limited the depth of individual experiences compared with one-to-one interviews. Participants may have felt reluctant to express sensitive or dissenting views in a group setting, particularly when discussing stigma-related attitudes. Second, the online interview format introduced potential technical challenges, including unstable internet connections and interruptions, which may have affected communication quality and the completeness of data collection. Third, all participants were recruited from a specific educational and cultural context, which may limit the transferability of the findings to other nursing student populations or healthcare disciplines.

Future studies may benefit from combining focus groups with individual interviews and incorporating face-to-face qualitative data collection where feasible. This would allow for a more comprehensive understanding of participants' experiences and improve the trustworthiness of the findings. Despite these limitations, this study provides important evidence supporting the feasibility and potential effectiveness of culturally tailored stigma-reduction interventions for nursing students and offers practical guidance for future large-scale implementation.

## Conclusion

This study explored the experiences of nursing students who participated in a pilot randomized controlled trial (RCT) of a culturally tailored intervention to reduce schizophrenia stigma. The findings showed that the intervention had good feasibility, high accessibility, and positive perceived efficacy in improving knowledge, attitudes, behavioral intentions, and empathy toward people with schizophrenia. Participants also provided valuable suggestions for improving intervention delivery and outcome measurement.

These findings support the use of culturally sensitive and empathy-based stigma-reduction interventions in nursing education. The study provides practical guidance for future full-scale RCTs and may inform the development of stigma-reduction programs for healthcare students and professionals. Improving attitudes and empathy toward people with schizophrenia is essential for reducing discrimination and promoting compassionate, person-centered mental health care.

## Data Availability

The raw data supporting the conclusions of this article will be made available by the authors, without undue reservation.
