# Supplementary material for: Nursing students' perceptions of an anti-stigma intervention for schizophrenia: a qualitative focus group study
Source: Front Public Health. 2026 Jun 25;14:1870693. doi: 10.3389/fpubh.2026.1870693 (PMC13348898; doi:10.3389/fpubh.2026.1870693)
Supplement: Supplementary file 1 [file Data_Sheet_1.PDF]

Appendix 1. Interview Guide

| | |
|---|---|
| 1 | How would you describe your overall experience of participating in the intervention program?<br>[Which week or part of the intervention did you like the most or least, and why? Were there any moments that were particularly memorable for you?] |
| 2 | Did you encounter any difficulties or challenges while completing the intervention program?<br>[For example, time commitment, workload, motivation, or emotional discomfort. Why do you think adhering to the intervention was easy or difficult? What factors influenced your participation and compliance?] |
| 3 | How did you feel about the frequency, duration, and overall arrangement of the intervention sessions?<br>[Do you think the intervention sessions were too frequent, too long, or appropriate? What do you think would be the optimal frequency and duration for nursing students?] |
| 4 | What do you think about the acceptability of the intervention program?<br>[Which parts of the intervention were easy or difficult to accept? What factors influenced your acceptance? What type of intervention format do you think would be more suitable for nursing students?] |
| 5 | How do you view the online delivery method of the intervention?<br>[Compared with face-to-face intervention, how effective do you think the online format was, and why? What were the advantages and disadvantages of online delivery?] |
| 6 | What do you think about the questionnaires and scales used in this study?<br>[Do you think these scales were appropriate for measuring knowledge, attitudes, empathy, and behavioural intentions toward people with schizophrenia? Why or why not? Were any questions unclear or difficult to answer?] |
| 7 | In what ways, if any, has participating in this program influenced your knowledge, attitudes, empathy, or behavioural intentions toward people with schizophrenia?<br>[What benefits have you gained from joining the program? Has the program changed how you understand or interact with people with schizophrenia? Can you share specific examples?] |
| 8 | What aspects of the intervention program do you think could be improved?<br>[What kind of intervention would be more suitable for nursing students? At what stage of nursing education do you think this intervention should be introduced, and why?] |

| | |
|---|---|
| 9 | Do you have any other suggestions or comments about the intervention program or this study that you would like to share? |
