# Supplementary material for: Nursing students' perceptions of an anti-stigma intervention for schizophrenia: a qualitative focus group study
Source: Front Public Health. 2026 Jun 25;14:1870693. doi: 10.3389/fpubh.2026.1870693 (PMC13348898; doi:10.3389/fpubh.2026.1870693)
Supplement: Supplementary file 2 [file Data_Sheet_2.PDF]

Appendix 2 The intervention implementation process

	Aim of the intervention	Intervention contents	Intervention methods and activities	Intervention time
First week	The intervention in the first week aimed to improve the knowledge and understanding of schizophrenia.	A. Knowledge about the aetiology, pathophysiology, clinical manifestations, treatment, nursing, and prognosis of schizophrenia<br>B. Differences between schizophrenia and other mental illnesses, such as multiple personality disorder, autism, depression, and psychopathy<br>C. Schizophrenia-related legal issues<br>D. The influence of cultural factors on the recognition of schizophrenia	In week 1, all five students in each subgroup worked as a team (collaborative learning activity) to investigate knowledge (investigative learning activity). They reported their findings through presentations, discussions, and debates (interactive learning activity). These groups of five students analysed, constructed, and evaluated the information to make sense of their acquired knowledge. The students were engaged in (higher-order thinking) activity when analysing this information.	Each group had 35 minutes to report, and 15 minutes for Q&A, discussion, and comment. The total duration was 2.5 hours.
Second week	The intervention in the second week aimed to correct the misconceptions about schizophrenia obtained from social media.	Hyperbolic reports about schizophrenia on the Internet and in literary works, novels, films, and TV plays	In week 2, groups of five students worked as a team (collaborative learning activity) to collect materials from the Internet, literary and artistic works, novels, films, and TV series with exaggerated reports about schizophrenia (investigative learning activity). Each group found 10 items containing exaggerated propaganda	Each group was required to find 10 items and explain them. Each group had 35 minutes to report, and 15 minutes for Q&A, discussion, and comment. The total

			about the abnormal behaviour and actions of people with schizophrenia (higher-order thinking). These groups also needed to establish the truth about misconceptions of schizophrenia and discuss or debate them (interactive learning activity).	duration was 2.5 hours.
Third week	The intervention in the third week aimed to contact people recovering from schizophrenia and psychiatric nurses.	Nursing students interacted with people recovering from schizophrenia. These people shared their experiences of the overall treatment and rehabilitation period. Nursing students had interactive communication with psychiatric nurses. The nurses shared their working experiences and their feelings about working in the psychiatric department.	In week 3, groups of five students worked as a team (collaborative learning activity) to prepare 10 questions (investigative learning activity) before meeting clients who were recovering from schizophrenia and psychiatric nurses. The clients and nurses shared their experiences and feelings to correct the students' misconceptions. Higher-order thinking skills were involved. There was Q&A, discussion, and debate during or after the interview (interactive learning activity).	The total duration was 3 hours, including the interview with two people recovering from schizophrenia and two psychiatric nurses, and a Q&A session.
Fourth week	The interventions in the fourth week involved self-reflection and concept mapping to deal with stigma.	Nursing students were required to undertake reflective critical and imaginative thinking autonomously on the following assigned topics<br>Nursing students were engaged in a	In week 4, nursing students were asked to write down their feelings (Subjects: 'If I suffered from schizophrenia, how would I live, get treatment, recover, and reintegrate, how would I face the	The total duration was 3 hours.

	An understanding of schizophrenia in Chinese traditional culture and religion, and the understanding and views of Buddhism, Taoism, and Confucianism regarding schizophrenia were achieved through nursing students' self-reflection and thinking.	teamwork exercise using a concept map to identify strategies to reduce schizophrenia stigma.	stigmatisation of schizophrenia by Chinese traditional culture and religion, and how would I feel when I encountered stigma?') (investigative learning activity), read other nursing students' opinions, and hold discussions or debates (interactive learning activity).<br>After that, groups of five students worked as a team (collaborative learning activity) to develop concept maps for the identified problems and solutions (higher-order thinking activity).	
